# Promotion of direct electron transfer between *Shewanella putrefaciens* CN32 and carbon fiber electrodes via *in situ* growth of α-Fe_2_O_3_ nanoarray

**DOI:** 10.3389/fmicb.2024.1407800

**Published:** 2024-06-13

**Authors:** Xiu He, Hao Lu, Jingjing Fu, Huang Zhou, Xingchan Qian, Yan Qiao

**Affiliations:** ^1^Institute of Basic Medicine and Forensic Medicine, North Sichuan Medical College, Nanchong, China; ^2^School of Materials and Energy, Southwest University, Chongqing, China; ^3^State Key Laboratory of Advanced Technology for Materials Synthesis and Processing, Wuhan University of Technology, Wuhan, China; ^4^Hubei Longzhong Laboratory, Xiangyang, China; ^5^Department of Chemistry, School of Pharmacy and Institute of Pharmacy, North Sichuan Medical College, Nanchong, China

**Keywords:** α-Fe_2_O_3_ nanoarray, direct electron transfer, microbial fuel cells, *Shewanella putrefaciens* CN32, iron transport system

## Abstract

The iron transport system plays a crucial role in the extracellular electron transfer process of *Shewanella sp*. In this study, we fabricated a vertically oriented α-Fe_2_O_3_ nanoarray on carbon cloth to enhance interfacial electron transfer in *Shewanella putrefaciens* CN32 microbial fuel cells. The incorporation of the α-Fe_2_O_3_ nanoarray not only resulted in a slight increase in flavin content but also significantly enhanced biofilm loading, leading to an eight-fold higher maximum power density compared to plain carbon cloth. Through expression level analyses of electron transfer-related genes in the outer membrane and core genes in the iron transport system, we propose that the α-Fe_2_O_3_ nanoarray can serve as an electron mediator, facilitating direct electron transfer between the bacteria and electrodes. This finding provides important insights into the potential application of iron-containing oxide electrodes in the design of microbial fuel cells and other bioelectrochemical systems, highlighting the role of α-Fe_2_O_3_ in promoting direct electron transfer.

## 1 Introduction

Microbial fuel cells (MFC) can convert the chemical energy of organic matter in sewage into usable electric energy, thereby generating clean energy while treating harmful pollutants in sewage. This technology can help alleviate the energy shortage caused by the depletion of fossil fuel supplies and address environmental pollution caused by fossil fuel combustion and wastewater treatment (Rikame et al., 2018; Tang et al., 2019; Zhai et al., 2019; Cheng et al., 2021). MFC represents a very promising new energy system that has garnered wide attention in the fields of wastewater treatment, biosensing, power generation, and intelligent wearables (Tang et al., 2019; Guo et al., 2021). The performance of MFC is usually limited by bioelectrocatalytic performance of the MFC anode, which not only provides a surface for bacterial adhesion but also acts as an electron collector (Sonawane et al., 2017; Liu et al., 2020). Therefore, it is crucial to design and develop suitable materials for MFC anodes that promote extracellular electron transfer and increase microbial loading.

Carbon fiber materials, such as carbon cloth, carbon paper, carbon felt, carbon brush, and graphite brush, are extensively used MFC anodes. However, their low surface energy, hydrophobic characteristics, and relatively smooth surfaces lead to lower microbial loads. To enhance the performance of MFCs, researchers have modified the surfaces of diverse carbon-based electrode with iron oxides. For example, Fe_2_O_3_ and Fe_3_O_4_ electroplated non-woven carbon fiber anode facilitated the immobilization of *S. oneidensis* MR-1 on the electrode and promoted biofilm formation, thereby improving the performance of the MFC (Phansroy et al., 2016). It has been demonstrated that Fe/Fe_2_O_3_ nanoparticles deposited on carbon felt, carbon cloth and graphite led to a significant increase in power generation, which attributed to the improved wettability of the electrode surface for enhanced adhesion between the microbial community and the modified electrode surface (Mohamed et al., 2018). Further, nanostructured Fe_2_O_3_ could improve the biocompatibility and provide large active surface area, which often resulted in the higher energy output of MFCs (Ge et al., 2013; Pandit et al., 2014). It has been reported that a hematite modified stainless steel anode exhibited enhanced bio-capacitance and charge-storage capacity, resulting in significant enhanced power density (Jadhav et al., 2015). The above reports reveal that iron oxide nanomaterials with various morphologies can enhance MFC performance mainly by reducing the internal resistance and increasing the adhesion of biofilm. However, the detail mechanism is still not very clear. It has been noted that the interaction between α-Fe_2_O_3_ and bacterial outer-membrane *c*-type cytochromes (OM *c*-Cyts) are posited to promote extracellular electron transfer (EET) processes (Nakamura et al., 2013; Liu et al., 2021) but more evidences are required.

In our prior research, heightened expression of genes associated with the iron transport system has been observed in a *Shewanella putrefaciens* CN32 (*S. putrefaciens* CN32) MFC with nickel oxide@carbon nanowire network decorated carbon cloth anode. It is possible that the iron transport system also play an important role in the interfacial electron transfer between *S. putrefaciens* CN32 and the α-Fe_2_O_3_. With this background, vertically aligned α-Fe_2_O_3_ were fabricated on carbon fiber cloth via a one-step hydrothermal method and subsequently used as anode in *S. putrefaciens* CN32 MFCs. The impact of Fe oxides on the surface properties of carbon cloth and electron transfer at the interface of *S. putrefaciens* CN32 were examined by analyzing their impact on facilitating the growth of anodic biofilm, catalyzing bioelectricity production, and influencing the expression of OM *c*-Cyt and iron system genes.

## 2 Experimental section

### 2.1 Materials synthesis

The carbon cloths were cut into strips measuring 2 cm × 4 cm, boiled in ultra-pure water for 30 min, and then placed in glass cups containing acetone, dilute hydrochloric acid, and ultra-pure water for ultrasonic cleaning, which lasted for 30 min. Subsequently, they were boiled in secondary water for an additional 30 min and air-dried at room temperature to prepare for the hydrothermal reaction. For the growth of α-Fe_2_O_3_ nanoarray on carbon cloth, 0.57 *g* of Na_2_SO_4_ and 1.1 *g* of FeCl_3_⋅6H_2_O were dispersed in 80 mL of ultra-pure water and mixed thoroughly under a magnetic stirrer until a clear solution was formed. The resulting solution was then transferred into a 100 mL polytetrafluoroethylene-lined reactor. After transferring the cleaned carbon cloth to the reactor, it was completely sealed, and the reactors were placed in an oven for different hours at varying reaction temperatures. After cooling the reactors down to room temperature sequentially, the samples were collected for further experiments. As the nanoarray structure only can obtained at 120°C (Supplementary Figure 1), the samples obtained at 120°C with different reaction time, denoted as α-Fe_2_O_3_@CC-1, α-Fe_2_O_3_@CC-2, and α-Fe_2_O_3_@CC-3 (for 2, 4, and 8 h, respectively), were used in following analyses. After hydrothermal reaction, the obtained samples were transferred into a tube furnace for annealing at 450°C for 2 h.

### 2.2 Physical characterizations

The morphology and structure of the samples were characterized using a Field Emission Scanning Electron Microscope (FESEM JSM-7800F JEOL). X-ray diffraction (XRD) patterns were acquired using an XRD-7000 instrument (SHIMADZU) with Cu Kα radiation at a scan rate of 2° per minute over a range of 5°–90°. The elemental composition and chemical states of the samples were analyzed with X-ray photoelectron spectroscopy (XPS) employing an Al Kα X-ray source on a Thermo Scientific K-Alpha+ system (Thermo Fisher Scientific).

### 2.3 MFC setup and operation

The MFC setup has been described in our previous work (Wu et al., 2018a). The α-Fe_2_O_3_@CC electrode was cut into 1 cm × 1 cm squares and used as the anode while the cathode consisted of a carbon fiber brush. Lactate, with a final concentration of 18 mM, served as the sole electron donor and was added to the anolyte (M9 buffer: Na_2_HPO_4_, 6 *g* L^–1^; KH_2_PO_4_, 3 *g* L^–1^; NH_4_Cl, 1 *g* L^–1^; NaCl, 0.5 *g* L^–1^; MgSO_4_, 1 mM; and CaCl_2_, 0.1 mM). The catholyte consisted of a 0.01 M phosphate buffer with 50 mM potassium ferricyanide. The MFC operated at room temperature (30°C) with an external resistance of 1.5 kΩ. The output voltage was measured using a digital multimeter. Once the MFC reached a steady state, polarization and power density curves were obtained by measuring the stable voltage output under various external resistances (1-80 kΩ). The surface morphology of the discharged anode was examined using FESEM in previous work (Qiao et al., 2015).

### 2.4 Electrochemical measurements

The electrolyte is buffered saline (PBS) buffer with 2 μM flavin mononucleotide (FMN) or an anaerobic M9 buffer supplemented with *S. putrefaciens* CN32 cell suspension. The α-Fe_2_O_3_@CC electrode was used as the working electrode, the titanium plate as the counter and saturated calomel electrode (SCE) as reference electrodes. The cyclic voltammogram (CV) was recorded between 0.8 and 0 V (vs SCE) with a scan rate of 5 mV s^–1^ or between -0.8 and 0.6 V (vs SCE) with a scan rate of 1 mV s^–1^ after continuous discharge 48 h under 0.2 V (vs SCE). Differential pulse voltammetry (DPV) was performed from −0.8 to 0 V (vs SCE) with a potential step of 4 mV, an amplitude of 25 mV, and a frequency of 1 Hz. Electrochemical impedance spectroscopy was carried out in a frequency range of 0.1 Hz to 100 kHz with a perturbation signal of 10 mV at −0.45 V.

### 2.5 Observation of bacterial staining on the electrode surface

To prepare a 0.85% NaCl solution, sterilize the solution and then aliquot 600 μL into each well of a 24-well plate to serve as a buffer. After evaluating the electrochemical behavior of the electrode, wash it thoroughly with the buffer solution before placing it in a well filled with the same buffer solution. Introduce 0.75 μL of each component—A and B—from the LIVE/DEAD BacLight Bacterial Viability Kit to the well containing the electrode. Incubate in the dark for 20 min to allow for effective staining. Upon completion of the staining process, gently rinse the electrode to remove excess dye, carefully transfer it to a new well containing 2 mL of the buffer solution, and allow it to rest for 10 min. During this period, agitate the plate gently and shield it from light to perform three rinsing cycles. Transfer the thoroughly rinsed electrode to a microscope slide. Use filter paper to delicately blot away any remaining buffer from one side of the electrode surface without directly touching the electrode. Finally, place the slide under a fluorescence inverted microscope for observation and documentation of the results.

### 2.6 Target genes selections and validation of target genes by real-time PCR

The core genes related to the iron transport system that was reported in our previous work (He et al., 2020) include heme ABC transporter ATP-binding protein (hABCATP), hemin ABC transporter substrate-binding protein (hemeABC), energy transducer TonB (enTonB), and iron ABC transporter permease (iron ABC). Additionally, the target genes encompass both the core genes related to the iron transport system and the outer membrane electron transfer-related genes (*mtrA*, *mtrB*, *mtrC*, *undA*, *feoA*, *feoB*) of *S. putrefaciens* CN32 (Wu et al., 2018b; Zou et al., 2019).

Total RNA was extracted from the bacterial biofilm near the α-Fe_2_O_3_@CC-3 anode of the MFC full battery running for 48 h, and from the bacterial biofilm on the α-Fe_2_O_3_@CC-3 electrode of the MFC full battery running for one week. Additionally, total RNA was extracted from the carbon cloth electrode as a control. Biological repeats were performed three times. Total RNA from cultures was extracted using RNAiso Plus (Takara Co., Dalian, China) according to the manufacturer’s protocol. After determining the quality of the extracted RNA, RNA was used to synthesize cDNA. The target gene expression level was determined by the 2^–ΔΔCt^ method and by three technical replicates, *recA* was used as a reference gene (Kim et al., 2017).

## 3 Results and discussion

**FIGURE 1:**
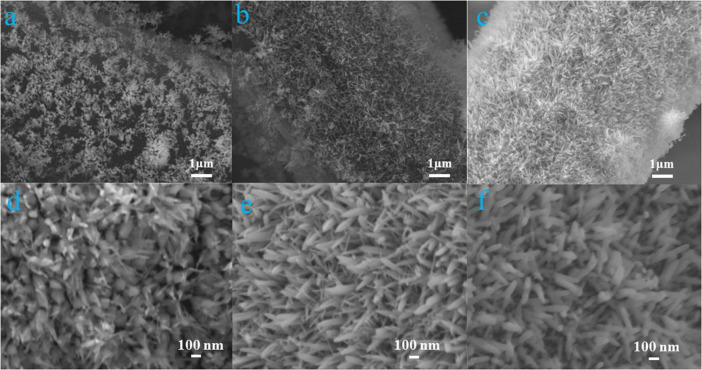
α-Fe_2_O_3_@CC-1 **(a,d)**, α-Fe_2_O_3_@CC-2 **(b,e)** and α-Fe_2_O_3_@CC-3 **(c,f)** FESEM images.

The morphologies of different α-Fe_2_O_3_@CC electrodes are shown in Figure 1. It can be observed that the α-Fe_2_O_3_ nanoarray progressively increase in thickness as the reaction time prolongs. The three α-Fe_2_O_3_@CC electrodes exhibit similar morphology with a uniform size distribution. The average diameter ranging from 20 to 80 nm and an average length ranging from 350 nm to 1 μm.

**FIGURE 2:**
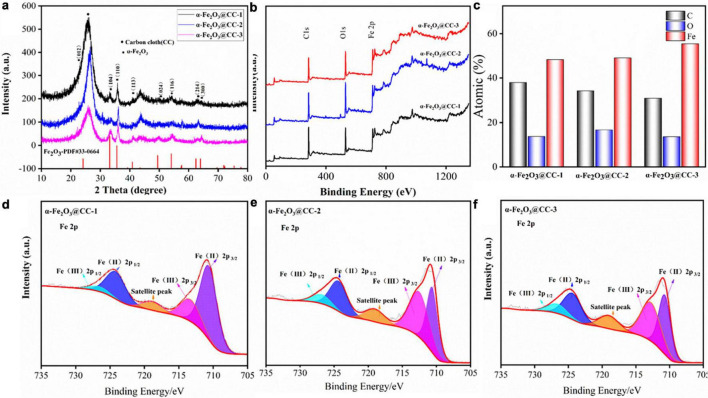
**(a)** XRD patterns of different α-Fe_2_O_3_@CC, **(b)** XPS total spectrum of different α-Fe_2_O_3_@CC, **(c)** different α-Fe_2_O_3_@CC With atomic ratio, **(d)** High-resolution Fe2p spectrums of α-Fe_2_O_3_@CC-1, **(e)** high-resolution Fe2p spectrums of α-Fe_2_O_3_@CC-2, **(f)** High-resolution Fe2p spectrums of α-Fe_2_O_3_@CC-3.

Various physical characterizations including XRD and XPS analyses were evaluated for the crystal and surface properties structure in different α-Fe_2_O_3_@CC electrodes. XRD results of three α-Fe_2_O_3_@CC samples are shown in Figure 2a, it can be seen that there is a very wide diffraction peak at 2 θ = 26°, which can be attributed to the presence of a carbon structure in the carbon cloth. The carbon peak of α-Fe_2_O_3_ materials, calcined at different hydrothermal times of 2, 4, and 8 h, significantly weakens with the increase of hydrothermal time. This observation suggests that the coverage of α-Fe_2_O_3_ on the carbon cloth increases as the reaction time extends. Furthermore, the main diffraction peaks of α-Fe_2_O_3_ are observed in the XRD pattern at θ values of 21°, 33°, 35°, 41.5°, 50°, 54.5°, 63°, and 64°. These diffraction peaks are consistent with the standard reference patterns of pure hematite in the PDF database (PDF # 33-0664) and correspond to the crystal planes (012), (104), (110), (113), (024), (116), and (300) of hematite (Jiao et al., 2014; Liu et al., 2021). Additionally, no obvious impurity peaks are observed in the XRD spectra.

The surface elements and their binding energies in the three α-Fe_2_O_3_@CC electrodes were analyzed using XPS, as shown in Figures 2b–f. Figure 2b displays the XPS total spectrum of the three α-Fe_2_O_3_@CC electrodes, revealing the presence of C, O, and Fe atoms. The observed chemical binding energies of 284.29, 530.08, and 710.41 eV correspond to the characteristic peaks of C 1s, O 1s, and Fe 2p, respectively. This confirms that the main elemental constituents of the nanoarray materials are C, O, and Fe, which is consistent with the XRD analysis results. Figure 2c demonstrates that iron content progressively increases as the reaction time extended. This trend may be attributed to the prolonged reaction time leading to the accumulation of α-Fe_2_O_3_ on the carbon cloth substrate. As can be seen from the Fe 2p spectrum shown in Figures 2d–f, three prominent peaks were observed. Two principal peaks are ascribed to the Fe 2p core-level electrons: Fe 2p3/2 and Fe 2p1/2, manifesting at binding energies of 711.18 eV and 724.78 eV, respectively. Additionally, a shake-up satellite peak is discernible at 719.18 eV across all three samples examined. The deconvoluted Fe 2p spectra indicate the presence of both Fe^3+^ and Fe^2+^ oxidation states, with Fe^3+^ peaks located at 713.41 eV for Fe 2p3/2 and 726.88 eV for Fe 2p1/2. Concurrently, Fe^3+^ peaks are observed at 710.81 eV for Fe 2p3/2 and 724.34 eV for Fe 2p1/2. These values are consistent with those reported in the literature for α-Fe_2_O_3_ (Orisekeh et al., 2021; Tamboli et al., 2022; Aalim and Shah, 2023). The comparative analysis of the Fe 2p spectra across the three samples indicates a remarkable similarity, suggesting the coexistence of Fe^2+^ and Fe^3+^ species within the α-Fe_2_O_3_ structure.

**FIGURE 3:**
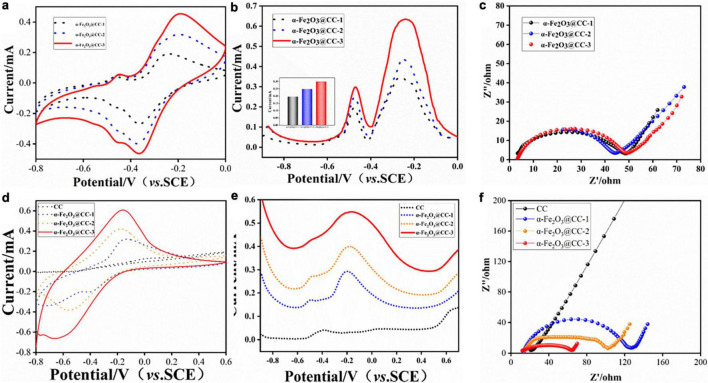
**(a–c)** Electrochemical behavior of different α-Fe_2_O_3_@CC in 0.1M PBS solution containing 2 μM FMN: **(a)** CVs, **(b)** DPVs, the inset is FMN redox peak current, **(c)** EIS curve. **(d–f)** Electrochemical behavior of different α-Fe_2_O_3_@CC electrodes in the *S. putrefaciens* CN32 bacterial suspension containing 18 mM lactate: **(d)** CVs, **(e)** DPVs, **(f)** EIS curve.

To evaluate the bioelectrocatalytic effects of flavins on three α-Fe_2_O_3_@CC electrodes, comprehensive electroanalytical techniques were employed. Cyclic voltammograms (CVs), differential pulse voltammograms (DPVs) and electrochemical impedance spectra (EIS) measurements were conducted in a 0.1 M phosphate buffer solution containing 2 μM FMN, utilizing a three-electrode system. Analysis of the CV and DPV profiles (Figures 3a, b) indicates an increase in both capacitance and redox peak currents for the α-Fe_2_O_3_@CC electrodes in proportion to the rise in α-Fe_2_O_3_ content. Each electrode exhibited a pair of modest redox peaks at −0.45 V, attributed to the redox processes of the flavin electron mediators (Zou et al., 2016). Riboflavin (RF) and flavin mononucleotide (FMN) play a pivotal role in facilitating extracellular electron transport, the increase of flavin secretion can improve the performance of MFC (Okamoto et al., 2014). A pronounced redox peak is noted at −0.3 V, corresponding to the Fe_2_O_3_/FeO redox couple. Further analysis of the DPV derived FMN reaction peak current (Figure 3b) suggests that the peak currents amplify concomitantly with the increase of α-Fe_2_O_3_ content in the electrodes. This enhancement is likely due to the augmented surface area provided by a higher density of α-Fe_2_O_3_ nanoarray, facilitating extensive active site engagement. The EIS Nyquist plots (Figure 3c) of the three electrodes are similar, which implies that the electrochemical behavior of the electrodes in the presence of flavins is concordance. Consequently, α-Fe_2_O_3_@CC-3 may manifest the most superior bioelectrocatalytic performance attributable to its higher content of α-Fe_2_O_3_.

To further investigate the bioelectrocatalytic behavior at α-Fe_2_O_3_@CC anode, the CVs, DPVs and EIS were conducted in *S. putrefaciens* CN32 MFC half-cells containing 18 mM lactate. The steady state CVs shown in Figure 3d illustrate an increase in redox peak current and double-layer capacitance for α-Fe_2_O_3_@CC electrodes, especially for α-Fe_2_O_3_@CC-3, suggesting enhanced capacitance and consistent with prior studies (Pandit et al., 2014; Jadhav et al., 2015). Flavin-mediated reactions were negligible in bacterial half-cells (Figure 3e), due to low flavin concentration and weak adsorption, but a redox peak around −0.3V indicated dominance of direct electron transfer via cytochromes in *S. putrefaciens* CN32 at α-Fe_2_O_3_@CC electrodes (Okamoto et al., 2011). The Nyquist plots of all three α-Fe_2_O_3_@CC electrodes had reduced interfacial electron transfer impedance compared to unmodified carbon cloth, with α-Fe_2_O_3_@CC-3 exhibiting the least resistance, confirming superior bioelectrocatalytic activity and potential for improved MFC performance. Post-analysis via live/dead bacterial staining and inverted fluorescence microscopy (Supplementary Figure 2) showed higher bacterial counts on α-Fe_2_O_3_@CC electrodes compared to carbon cloth, with α-Fe_2_O_3_@CC-3 having the highest live bacteria count, indicating α-Fe_2_O_3_ promotes bacterial adsorption and biofilm growth, aligning with previous findings(Ge et al., 2013; Phansroy et al., 2016; Mohamed et al., 2018).

**FIGURE 4:**
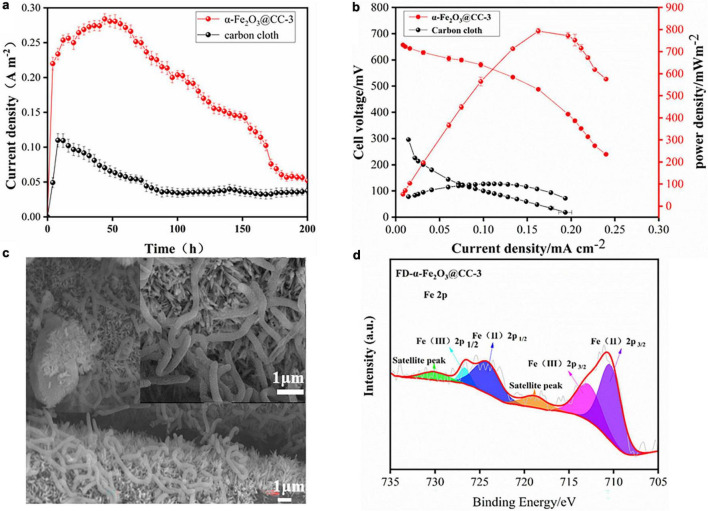
**(a)** α-Fe_2_O_3_@CC and carbon cloth electrodes one-round discharge curve; **(b)** α-Fe_2_O_3_@CC-3 and carbon cloth power curve; **(c)** α-Fe_2_O_3_@CC-3 The growth of biofilm after one round of discharge. **(d)** High-resolution Fe2p spectrums of α-Fe_2_O_3_@CC-3 electrode after one round of discharge.

To assess the bioelectrocatalytic performance of α-Fe_2_O_3_@CC-3 and carbon cloth anodes, they were tested in *S. putrefaciens* CN32 MFCs with the H-type two-chamber dual-chamber configuration. The α-Fe_2_O_3_@CC-3 anode achieved a maximum current density of 0.28 mA cm^–2^, doubling that of carbon cloth anode (0.10 mA cm^–2^), and exhibited a maximum power density of 816 mW m^–2^, which is eight-fold higher than that of the carbon cloth anode (101 mW m^–2^, Figure 4). It is four times higher than another reported MFC with iron oxide nanoparticles modified carbon cloth anode (Mohamed et al., 2018). The performance of the α-Fe_2_O_3_@CC-3 anode is also superior to other porous or nanostructured anodes reported in our previous work (Supplementary Table 2). These results indicate that the α-Fe_2_O_3_@CC-3 anode substantially enhances MFC performance. Post-discharge analysis using FESEM revealed denser bacterial colonization on α-Fe_2_O_3_@CC-3 anode compared to carbon cloth anode (Figure 4c and Supplementary Figure 3). The inclusion of α-Fe_2_O_3_ was anticipated not only to bolster biocompatibility but also to increase the current output (Long et al., 2019). Fe 2p XPS spectrum analysis post-discharge (Figure 4d) showed a decrease in Fe^3+^ peaks and an increase in Fe^2+^ peak intensity, suggesting Fe^3+^ to Fe^2+^ conversion on the α-Fe_2_O_3_@CC-3 electrode. (Orisekeh et al., 2021; Tamboli et al., 2022; Aalim and Shah, 2023) This may be linked to direct bacterial interaction, potentially influencing bacterial metabolism and gene expression.

**FIGURE 5:**
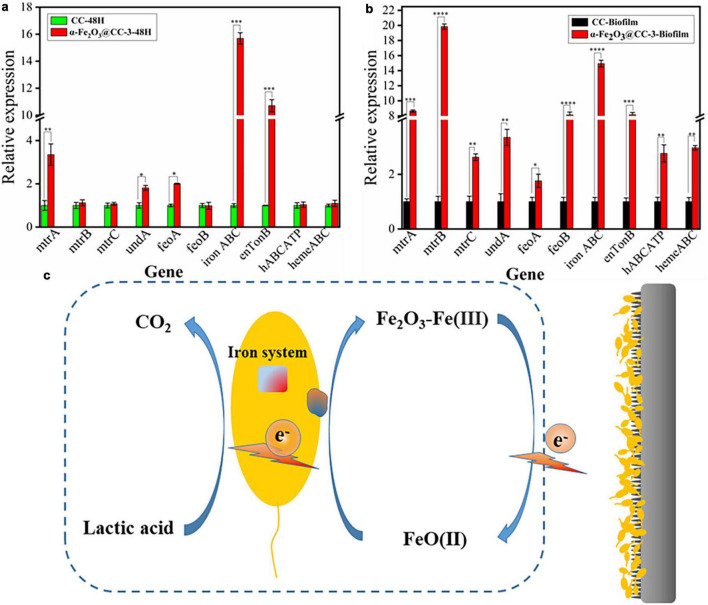
**(a)** qPCR analysis of target genes in *S. putrefaciens* CN32 MFC (CC and α-Fe_2_O_3_@CC-3 electrode) for 48 h. **(b)** qPCR analysis of target genes in *S. putrefaciens* CN32 MFC biofilm (CC and α-Fe_2_O_3_@CC-3 electrode). **(c)** Catalytic activity of α-Fe_2_O_3_ electrode.

At last, the impact of α-Fe_2_O_3_@CC anodes on gene expression in MFCs was investigated. RNA was extracted from both the electrode biofilm and solution after varying discharge times, and subsequently quantified through quantitative polymerase chain reaction (qPCR), with the resulting data being presented in Figure 5. In *S. putrefaciens* CN32, the gene clusters implicated in encoding the OM *c*-Cyts proteins are comprised of *feoA*, *feoB*, *undA*, *mtrC*, *mtrB*, and *mtrA*. In the vicinity of the α-Fe2O3@CC-3 electrode, notable upregulation of certain membrane genes (*mtrA*, *undA*, *feoA*) was observed in comparison to the carbon cloth electrode, while others (*mtrB*, *mtrC*, *feoB*) remained unchanged (Figure 5a). Moreover, iron transport genes (*enTonB, ironABC*) exhibited significant upregulation, indicative of active electron transport by bacteria at the α-Fe_2_O_3_@CC-3 anodee. This heightened expression was mirrored in the biofilm on the α-Fe_2_O_3_@CC-3 anode (Figure 5b). Functional genes related to iron system include FeO, a transporter functioning to specifically acquire Fe^2+^ from environments, ABC superfamily, TonB superfamily, and the ferric uptake regulator (Fur) etc. (Liu et al., 2018, 2022). In this work, *feoA*, *feoB*, *hABCATP*, *hemeABC*, *enTonB*, *iron ABC* are significantly up-regulated in biofilms on α-Fe_2_O_3_ anode, which indicated its beneficial to the absorption and utilization of iron by *Shewanella putrefaciens* CN32. Additionally, outer membrane *c*-type cytochromes, crucial for *Shewanella* species’ extracellular electron transfer (EET) during electrode respiration (Okamoto et al., 2014; Wu et al., 2018a; Zou et al., 2019), were also affected by the iron content since iron serves as the active center for proteins involved in various electron-generating and electron-transferring processes, such as cytochromes, dehydrogenases, and reductases. The ability of microorganisms to adsorb iron is particularly important in facilitating efficient electron transfer (Zhao et al., 2022). The promotion of direct electron transfer by α-Fe_2_O_3_ nanoarray was substantiated by the upregulated expression of iron transport genes and the enhanced bacterial electron transfer. The bacterial Fe system channeled electrons from lactate to the electrode, leading to the reduction of Fe_2_O_3_ to FeO. Subsequently, these electrons transitioned to the external circuit, where FeO reoxidized to Fe_2_O_3_, thereby facilitating electron transfer (Figure 5c). Notably, α-Fe_2_O_3_ may serve as an electron shuttle in the direct electron transfer scheme between bacteria and the anode.

As discussed above, α-Fe_2_O_3_ nanoarray anode demonstrates the potential to effectively upregulate the expression of Fe system genes and outer membrane genes in *Shewanella putrefaciens* CN32, serving as a conduit for electron transfer between electrodes and bacteria. Subsequent research endeavors could delve into the exploration of the Fe system genes of *Shewanella putrefaciens* CN32 and the cultivation of high-efficiency, electricity-generating strains through genetic manipulation. Despite the demonstrated enhancement in MFC performance attributed to the incorporation of α-Fe_2_O_3_ nanoarray, there remains scope for advancement in power density, highlighting the need for refinement in the morphology of α-Fe_2_O_3_.

## 4 Conclusion

A vertically oriented α-Fe_2_O_3_ nanoarray was fabricated on carbon cloth to enhance interfacial electron transfer in *S. putrefaciens* CN32 microbial fuel cells. The incorporation of the α-Fe_2_O_3_ nanoarray significantly enhanced biofilm loading, leading to an eight-fold higher maximum power density compared to plain carbon cloth. Through expression level analyses of electron transfer-related genes in the outer membrane and core genes in the iron transport system, we propose that the α-Fe_2_O_3_ nanoarray may serve as an electron mediator, facilitating direct electron transfer between the bacteria and electrodes. This finding provides important insights into the potential application of iron-containing oxide electrodes in the design of microbial fuel cells and other bioelectrochemical systems.

## Data availability statement

The original contributions presented in this study are included in the article/Supplementary material, further inquiries can be directed to the corresponding authors.

## Author contributions

XH: Conceptualization, Data curation, Formal analysis, Funding acquisition, Investigation, Methodology, Project administration, Resources, Software, Supervision, Validation, Visualization, Writing – original draft, Writing – review and editing. HL: Data curation, Formal analysis, Methodology, Software, Writing – review and editing. JF: Formal analysis, Funding acquisition, Software, Writing – review and editing. HZ: Data curation, Funding acquisition, Project administration, Writing – review and editing. XQ: Conceptualization, Project administration, Software, Writing – review and editing. YQ: Conceptualization, Data curation, Formal analysis, Funding acquisition, Investigation, Methodology, Project administration, Resources, Software, Supervision, Validation, Visualization, Writing – original draft, Writing – review and editing.
