# Supplementary material for: Promotion of direct electron transfer between Shewanella putrefaciens CN32 and carbon fiber electrodes via in situ growth of α-Fe2O3 nanoarray
Source: Front Microbiol. 2024 Jun 13;15:1407800. doi: 10.3389/fmicb.2024.1407800 (PMC11208625; doi:10.3389/fmicb.2024.1407800)
Supplement: Supplementary file 1 [file Data_Sheet_1.PDF]

Supplementary Information

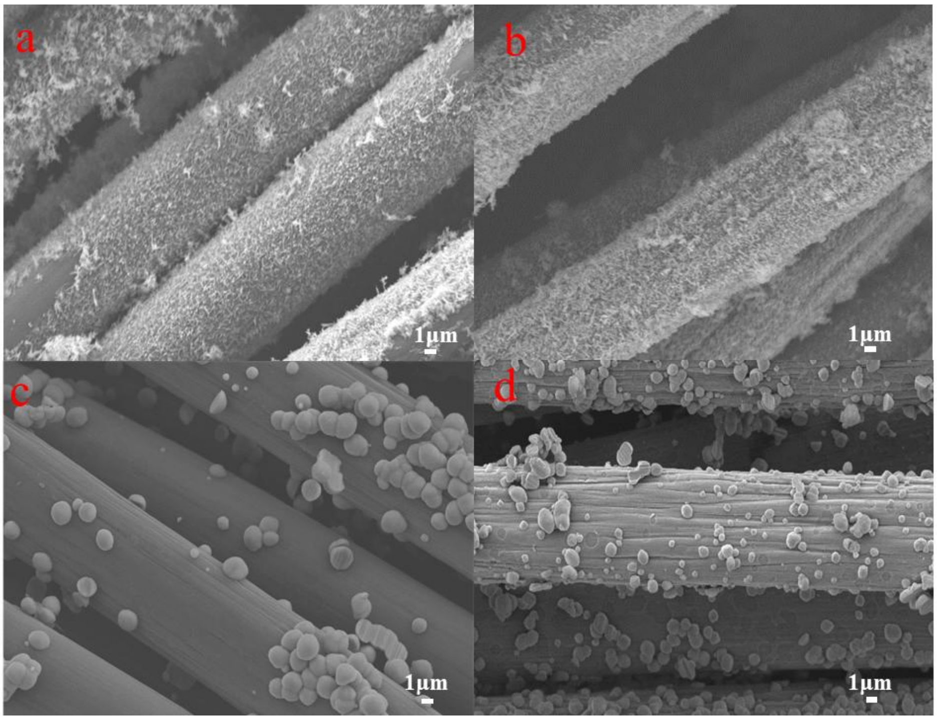

Figure S1 FESEM images of α-Fe_2O_3 at different temperatures (a-d, 90 ℃, 120 ℃, 150 ℃, 180 ℃).

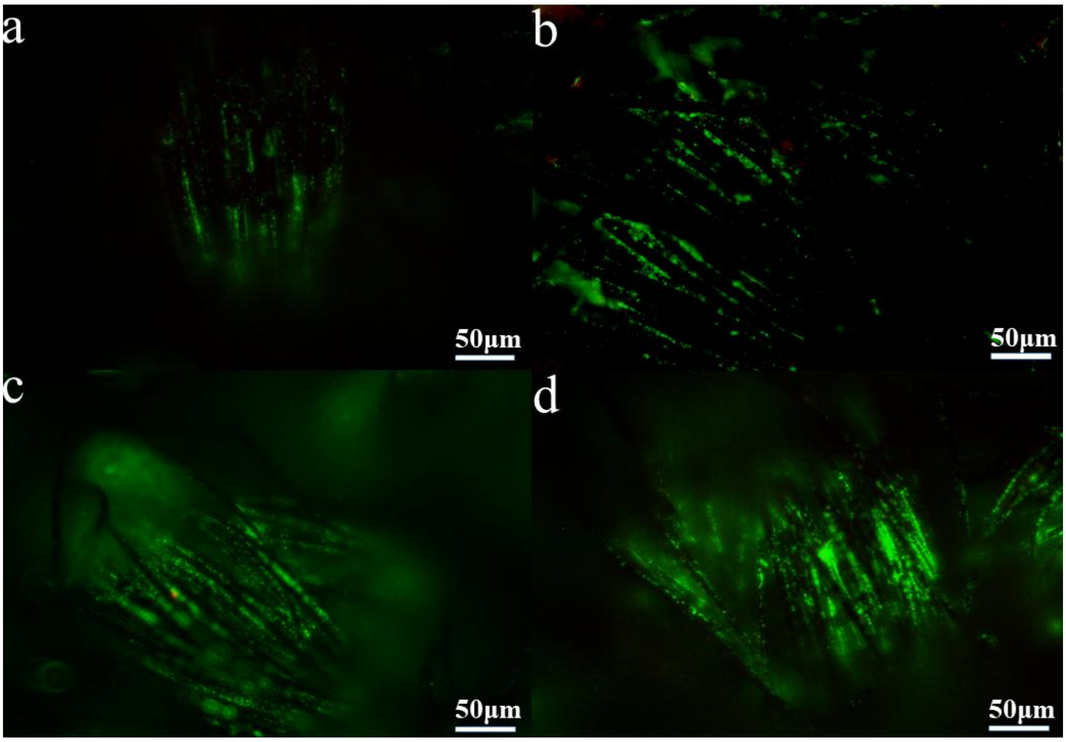

Figure S2 Electrode live and dead staining image (a) CC, (b) α-Fe_2O_3@CC-1, (c) α-Fe_2O_3@CC-2, (d) α-Fe_2O_3@CC-3.

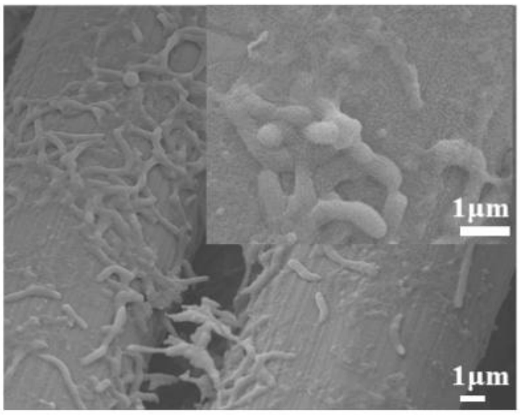

Figure S3 carbon cloth electrode after one-round discharge biofilm growth.

Table S1 Real-time fluorescence quantitative verification primers.

Gene	Forward primer (5'-3')	Reverse primer (5'-3')	Tm(°C)
mtrB	ATGATGTTAGCAGAGCCTGT	TCAAGTGCCATAGTGC	58
mtrA	CACTTGCCACAATCCTCACG	ACCTCCATGACATTGC	60
feoA	AAGCGAACTTAGTCCCGGTG	TGGGCGCTCGTCTAAT	58
feoB	GAACGCCATCTATATCTTACC	GCACACGCCAATGACA	60
mtrC	GCAGCGACAATAAGCATACA	CAACACGTTGGATCTG	60
undA	GCCGATAGTGGTTGTAGCAG	TTGCCGCAGTCAGTGA	60
hABCATP	CGGCATTATCGGCGTATCATC	GCAATAGGAGCACGAC	56
hemeABC	CGTGAGCGCCGAGCAAGATA	GCGGAGAGCATTCTGT	60
enTonB	ATCAGGCGCAGTTACGGCAT	GTGTTGTGGCAGTACC	60
ironABC	GAACCGAGGTTAGCCGATGC	CCGCCATTTCAGTCTT	56

Table S2 Maximum power density of reported MFCs with nanostructure or porous materials modified carbon cloth anode.

Anode	Cathode	Microbial catalyst	Maximum power density (mW m^{-2})
porous carbon–silica	carbon fiber brush	*S. putrefaciens* CN32	580.7 (Wu et al., 2018)
N-Doped reduced graphene oxide	carbon fiber brush	*S. putrefaciens* CN32	442.26 (Wu et al., 2018)

cellulose-derived porous carbon	carbon fiber brush	*S. putrefaciens* CN32	446 (Wang et al., 2020)
nitrogen-doped carbon nanowires	carbon fiber brush	*S. putrefaciens* CN32	510 (Wu et al., 2020)
Fe/Fe_2O_3 nanoparticle	carbon felt	Mixed bacteria	200 (Mohamed et al., 2018)

REFERENCES

Wu, X., Qiao, Y., Shi, Z., & Li, C. M. (2018). Enhancement of interfacial bioelectrocatalysis in Shewanella microbial fuel cells by a hierarchical porous carbon–silica composite derived from distiller's grains. *Sustainable Energy & Fuels*, 2(3), 655-662

Wu, X., Qiao, Y., Shi, Z., Tang, W., & Li, C. M. (2018). Hierarchically porous N-doped carbon nanotubes/reduced graphene oxide composite for promoting flavin-based interfacial electron transfer in microbial fuel cells. *ACS applied materials & interfaces*, 10(14), 11671-11677.

Wang, D., Wang, Y., Yang, J., He, X., Wang, R. J., Lu, Z. S., & Qiao, Y. (2020). Cellulose aerogel derived hierarchical porous carbon for enhancing flavin-based interfacial electron transfer in microbial fuel cells. *Polymers*, 12(3), 664

Wu, X., Qiao, Y., Guo, C., Shi, Z., & Li, C. M. (2020). Nitrogen doping to atomically match reaction sites in microbial fuel cells. *Communications Chemistry*, 3(1), 68

Mohamed, H. O., Obaid, M., Poo, K.-M., Ali Abdelkareem, M., Talas, S. A., Fadali, O. A., Chae, K.-J. (2018). Fe/Fe_2O_3 nanoparticles as anode catalyst for exclusive power generation and degradation of organic compounds using microbial fuel cell. *Chemical Engineering Journal, 349*, 800-807. doi:10.1016/j.cej.2018.05.138
